# Awareness of comfort immediately after a relaxation therapy session affects future quality of life and autonomic function: a prospective cohort study on the expectations of therapy

**DOI:** 10.1186/s13030-018-0135-y

**Published:** 2018-11-02

**Authors:** Hideaki Hasuo, Kenji Kanbara, Hiroko Sakuma, Mikihiko Fukunaga

**Affiliations:** Department of Psychosomatic Medicine, Kansai Medical University, Shinmachi 2-5-1, Hirakata, Osaka, 573-1090 Japan

**Keywords:** Awareness of a feeling of comfort, Expectation, Caregiver, Relaxation, Coping skill, Resonant breathing, Heart rate variability, Biofeedback, Quality of life, Autonomic function

## Abstract

**Background:**

High expectations regarding therapy are reported to have positive effects on future therapeutic course and related behavior. Some individuals are aware of feelings of comfort immediately after a relaxation therapy session.

**Methods:**

Heart rate variability biofeedback (HRV-BF) therapy using a relaxation technique called resonant breathing was administered to 44 family caregivers who felt burdened by their work caring for family members with cancer. We prospectively evaluated how the level of comfort participants were aware of immediately after an initial therapy session affected their expectations regarding the therapy, as well as future quality of life (QOL) and autonomic function. This study was a secondary analysis of a randomized, open-label study titled “Self-care system for family caregivers of cancer patients using resonant breathing with a portable home device”.

**Results:**

Among the participants, 56.8% were aware of a feeling of comfort immediately after an initial therapy session. Participants were then divided into two groups according to the presence or absence of their awareness of comfort. Expectation levels regarding the therapy were significantly increased in the awareness group after the therapy session (*P* = 0.003). No main effect between groups was observed for heart rate variability (HRV) during therapy (*P* = 0.949). Four weeks after the initial therapy session, QOL improved and HRV increased in the awareness group (*P* < 0.001).

**Conclusions:**

Better outcomes in the awareness group were not associated with HRV during therapy. A feeling of comfort immediately after a therapy session may have positive effects on future QOL and autonomic function by raising participants’ expectations of the therapy.

**Trial registration:**

UMIN000021639. Registered 27 March 2016

## Background

Several previous studies have reported that caregivers of family members with cancer are often deeply distressed, with reduced quality of life (QOL) and a higher prevalence of psychiatric disorders [1, 2]. Among family caregivers providing palliative care to cancer patients at home, QOL is often related to self-efficacy, and their coping skills are often decreased [3]. Specialized palliative care from an early stage has been reported to contribute to improved QOL for both cancer patients and family caregivers, by aiding the development of coping skills [4].

Biofeedback (BF) therapy is a coping technique in which physical and mental functions are regulated by measuring usually unnoticeable physiological information and feeding it back to patients via visualization. Heart rate variability biofeedback (HRV-BF) therapy using resonant breathing has been studied in depth [5], and its efficacy has been demonstrated in the treatment of autonomic hyperactivity, mood disorder, and psychosomatic disorders [6, 7]. HRV is a measure of the fluctuation in the interval between heartbeats, and reflects autonomic nerve activity. A low-frequency (0.04–0.15 Hz) component and a high-frequency (0.15–0.4 Hz) component are observed within several minutes of HRV [8]. These components are obtained via frequency domain analysis, and reflect parasympathetic activity. Resonant breathing is a relaxation technique in which patients breathe at a specific rate to maximize their HRV, an index of autonomic nervous system function. Using this technique, patients can increase their HRV by triggering the resonance between their breath and pressoreceptor reflex, which involves low-frequency components [5].

Because most family caregivers experience guilt about their ability to cope, they typically have limited capacity to employ coping techniques [9, 10]. A study of mindfulness-based stress reduction therapy among lung cancer patients and their family caregivers reported that, although the level of mental distress of cancer patients was reduced, the level of distress of caregivers was not reduced because they prioritized patients’ well-being over their own [11]. Therefore, new approaches to raising caregivers’ expectations regarding coping techniques could be a valuable component of specialized palliative care from an early stage.

Having high expectations of a therapy has been found to have beneficial effects on future course and behaviors, and an association between positive expectations and better health outcome was reported by a systematic review [12]. One study reported that the presence of positive expectations of a pain therapy before the actual therapy exerted a positive effect on the future course of pain and related behaviors [13]. In addition, the presence of high expectations for initial chemotherapy treatment reduced the number of adverse events [14]. Thus, the level of expectations of therapy appears to be an important factor in relation to subsequent placebo effects [15].

Few studies have examined potential factors that could raise the level of expectation of therapy. One previous study of patients with chronic lumbago (lower back pain) reported that placebo had positive effects on the course of pain even though patients were informed that the medicine was a placebo, but the validity of the placebo effect was concurrently explained to the patients [16]. Among these patients, receiving information from a trained medical professional about the efficacy of placebo treatment resulted in patients’ raised expectations regarding the treatment. Thus, the involvement of medical professionals may represent an expectation-raising factor. A number of clinical studies have reported that patients are aware of physiological changes in their body during relaxation therapy performed as a mental/physical coping technique. Relaxation techniques, including particular breathing methods, can induce psychological changes in the central nervous system, as well as physiological changes in peripheral tissues (e.g., blood vessels, saliva, skeletal muscles, body temperature, and heart rate) [6, 7, 17–19]. We hypothesized that patient awareness immediately after a relaxation therapy session would function as a patient-related factor, influencing the patients’ level of expectation regarding the therapy. To the best of our knowledge, no previous studies have tested this hypothesis to examine the effects of prior feelings on the future therapeutic course and related behaviors.

## Methods

### Study participants and eligibility criteria

This study was conducted between 2015 and 2017 at the Kansai Medical University Hospital. The study participants were 44 family caregivers caring for a family member with cancer, all of whom had HRV-BF with resonant breathing. In the current study, a family caregiver was defined as a family member who was directly providing care to a relative with cancer, including a spouse. The selection criteria included the caregiver reporting that they felt the burden of administering long-term nursing care. The level of nursing-care burden felt by the caregiver was evaluated using the Japanese version of the Zarit Caregiver Burden Interview (J-ZBI). Participants with a J-ZBI score ≥ 24, which is the cutoff value for the risk of depression, were eligible for this study [20]. Participants satisfying the following two items were excluded from the study: 1) having diseases that affected the evaluation of autonomic nerve function, such as diabetes; and 2) having any comorbidity relating to psychiatric diseases or conditions that made communication difficult, such as cognitive impairment, or schizophrenia.

### Study design

In our preliminary open-label randomized controlled study, 54 family caregivers caring for a family member with cancer, who had HRV-BF, were randomized into either a home self-care group (*n* = 27) or a control group (*n* = 27), according to whether they did the resonant breathing method using a portable device at their home [21]. The home self-care group did resonant breathing every day before bedtime, synchronizing their breath with the breath pacer included in the portable device. The control group did not use any particular method, including resonant breathing, before bedtime. The family caregivers in this study learned to quickly administer resonant breathing using a portable device at home, and resonant breathing improved rapidly, along with autonomic nerve function and quality of life. In the current observational study, we conducted a secondary analysis of this preliminary study data, prospectively evaluating the effects of awareness of a comfortable feeling immediately after an HRV-BF therapy session that used resonant breathing as a mental/physical coping strategy, on the level of expectation for the therapy as well as future QOL and autonomic function. For this study, we applied to the ethics committee to do a secondary analysis from the 11th case of the previous study.

### Measures

**Fig. 1:**
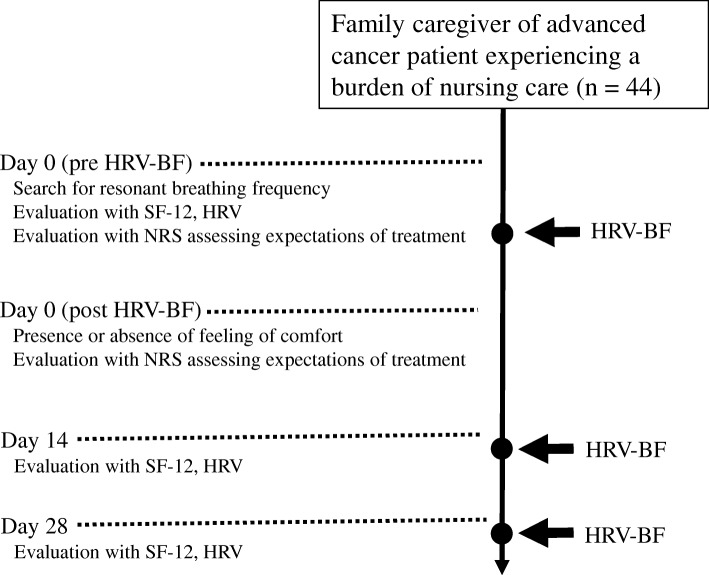
Flowchart showing the study procedure. *HRV* heart rate variability, *BF* biofeedback, *SF-12* 12-Item Short-Form Health Survey, *QOL* quality of life, *NRS* numerical rating scale

Figure 1 shows a flow chart of the experimental procedure. HRV-BF using resonant breathing was administered to caregivers at our medical institution for up to 30 min on day 0 and days 14, and 28 from the start of the intervention period). During HRV-BF, caregivers used the Breath Pacer application (ProComp Infiniti™/BioGraph Infiniti, Thought Technology Ltd., New York, USA) to maximize the HRV waveform displayed on the screen of a tablet personal computer (iPad mini; Apple, Cupertino, CA, USA). We connected the HRV components (myBeat WHS-2; Union Tool Co., Tokyo, Japan) to a special electrode pad attached directly to the participant’s chest. The HRV waveform was displayed on the screen in real time. Data entry with the Breath Pacer was carried out based on frequency.

At the start of the intervention (day 0), we monitored breathing among participants in both groups at our hospital, using a multichannel biofeedback system (ProComp Infiniti™/BioGraph Infiniti; Thought Technology Ltd., New York, USA), to determine the resonant breathing requency of the caregivers. Participants breathed for 2 min at 5, 5.5, 6, 6.5, and 7 breaths per minute while we measured breathing frequency and heart rate. We calculated the resonant breathing frequency as the number of breaths that maximized the LF and LF spectrum peak.

The participants’ level of expectation regarding the therapy was evaluated using a numerical rating scale (NRS) before and after the therapy session at day 0. According to the presence or absence of the awareness of a feeling of comfort after the therapy session at day 0, participants were divided into an awareness (of a feeling of comfort) group or a non-awareness (of a feeling of comfort) group. The questionnaire contained the following question: “Were you aware of a feeling of comfort or discomfort?” Participants who answered “somewhat” or “a little” were included in the non-awareness group.

Measurement of health-related QOL and HRV was done before HRV-BF (on days 0, 14, and 28). We evaluated the health-related QOL of family caregivers using the 12-Item Short-Form Health Survey (SF-12), a self-report questionnaire (on days 0, 14, and 28). The primary outcome of this study was the change rate in NRS, which was used to assess the difference in the expectations of treatment from pre HRV-BF to post HRV-BF, of the awareness and non-awareness groups. We measured HRV among all family caregivers prior to administering HRV-BF. Caregivers performed resonant breathing for 5 min, then rested for 5 min. We measured the low- and high-frequency power of HRV continuously for 15 min on days 0, 14, and 28. We used the HRV analysis software Kubios HRV version 3.1(Kubios Oy, Kuopio, Finland), which is reported to be highly reliable for short-term recording [22, 23].

The primary endpoint of this study was the difference between groups in the level of expectation regarding the therapy at Day 0. A secondary end point was the difference between groups of the low-frequency on Day 28.

#### Resonant breathing implementation rate at home

We measured the resonant breathing implementation rate at home as an evaluation of related behavior. We calculated this rate by dividing the number of days on which at-home family caregivers in the self-care group performed resonant breathing at home over the intervention period (28 days). The caregiver was instructed to do resonant breathing at home according to the Breath Pacer application for at least 5 min before going to bed each day.

#### NRS assessing expectation for treatment

Patients’ expectations regarding treatment-related improvements in QOL were determined via a questionnaire with a 0–10 NRS (0 = no improvement to 10 = full improvement) pre HRV-BF on day 0 and again post HRV-BF on day 0. The questionnaire contained the following question; “How much do you think your QOL will improve?” The questionnaire was self-administered. The validity of this questionnaire is not clear, but it has often been used in previous research [12, 13].

#### 12-item short-form health survey (SF-12)

The SF-12 is a health-related QOL scale that is not limited to assessment of patients with specific diseases. The reliability and validity of the SF-12 were established in a previous study [24]. We obtained three summary scores: a physical component summary (PCS), a mental component summary (MCS), and a role/social component summary (RCS) based on the participants’ responses to the 12 questions. Based on national standard values, we translated each summary score to a rating score (0–100 points). We determined phealth-related QOL by comparing the score with the national standard value (national standard value = 50 points; standard deviation = 10 points).

#### Heart rate variability (HRV)

HRV is a measure of the fluctuation in the interval between heartbeats, and reflects autonomic nerve activity. Resonant breathing is a method of breathing to achieve a frequency that maximizes HRV. Resonant breathing gives rise to a resonance between breathing and the baroreceptor reflex involving the low-frequency power of HRV, to increase HRV [5]. The mean value for resting HRV in adults is a high-frequency power of 657 ms^2^ [25].

### Statistical analysis

All data were reported as means with standard deviation, 95% confidence interval (95% CI), ranges, or frequencies (%), as appropriate. For all study participants, we calculated the rate of perceived feeling of comfort after the initial therapy session. For the group assigned to resonant breathing at home, we calculated the breathing implementation rate. Unpaired t-tests and Pearson’s chi-square test were used to analyze the dependent variables: age, sex, resonant frequency, resonant breathing at home, NRS assessment of the expectations of treatment, J-ZBI score, SF-12 score, and HRV score. When a participant withdrew from the study, the SF-12 and HRV scores after withdrawal were substituted with the score immediately before withdrawal.

The change in the course of NRS score used to assess expectations of treatment (day 0; pre HRV-BF, post HRV-BF), SF-12 score,and HRV score (day 0, day 14, day 28) were analyzed using one-way repeated measures analysis of variance (ANOVA) for each group. For comparisons between groups, we used time-course as the within-subjects factor and group as a between-subjects factor in two-way repeated measures ANOVAs. In the analysis of variance, multiple comparisons were corrected for using the Bonfferroni method.

Multivariable logistic regression analysis was performed with increased expectation of treatment after HRV-BF as a dependent variable, and seven independent variables, including age, sex, resonant frequency, awareness of a feeling of comfort, J-ZBI score, low-frequency power (day 0, pre HRV-BF), low-frequency power (day 0, post HRV-BF). Increased expectation of treatment after HRV-BF was determined as ≥8 or ≥ 33% improvement in NRS before and after HRV-BF.

A *P*-value of less than 0.05 was regarded as statistically significant. Statistical procedures were conducted using SPSS version 18.0 J for Macintosh (SPSS, Inc. IBM, Chicago, IL).

## Results

HRV-BF therapy using resonant breathing was administered at a medical institution. The results revealed that 56.8% (*n* = 25) of the participants were aware of a feeling of comfort after an initial therapy session. No participants reported the awareness of discomfort after the session. According to the presence or absence of the feeling of comfort, participants were divided into an awareness group (n = 25) and a non-awareness group (*n* = 19). The demographic data and clinical characteristics of both groups are shown in Table 1. In the awareness group, the RCS scores of the SF-12 were significantly lower. Although the awareness group tended to report a heavier care burden and lower low-frequency power, no significant differences were observed compared with the non-awareness group. Two participants (one from each group) withdrew from the study between day 14 and 28 because of the condition of the cancer patients they were caring for (completion rate in awareness group; 96.0%: non-awareness group; 94.7%). The implementation rate of resonant breathing at home was 95.7% in the awareness group (*n* = 13) and 76.4% in the non-awareness group (*n* = 11), significantly higher in the awareness group than in the non-awareness group (*P* < 0.007).

**Table 1:** Demographic and clinical characteristics of study participants

	Awareness group (*n*=25)		Non-awareness group (*n*=19)		*p*
Age, year	Mean	SD	Mean	SD	
	61.4	12.6	65.3	10.6	0.277
Sex	*n*	%	*n*	%	
Male	8	32	8	42	0.119
Female	17	68	11	58	
Relationship with the patient	*n*	%	*n*	%	
Mother	0	0	2	10.5	
Hasband	7	28	8	42.1	
Wife	15	60	8	42.1	
Son	1	4	0	0	
Daughter	2	8	1	5.3	
Resonant frequency	Mean	SD	Mean	SD	
	6.5	0.7	6	0.7	0.869
	*n*	%	*n*	%	
5	4	16	2	10.5	
5.5	7	28	2	10.5	
6	2	8	1	5.3	
6.5	10	40	4	21	
7	2	8	10	52.6	
Home self-care group (randomized allocation in the previous study)	*n*	%	*n*	%	
	13	52	11	57.8	0.705
NRS assessing expectations of treatment	Mean	SD	Mean	SD	
Before HRV-BF	6.4	1.9	6.3	1.8	0.6
After HRV-BF	8.6	1.4	6.8	2.2	0.004
J-ZBI	Mean	SD	Mean	SD	
	39.6	16.1	32.3	12.4	0.095
SF-12 (day 0)	Mean	SD	Mean	SD	
PCS	35.2	14.4	39.7	20.3	0.116
MCS	35.6	10.8	34.5	13.4	0.458
RCS	21.2	17.2	29.1	12.3	0.01
low-frequency power (day 0, resonant breathing)	Mean	SD	Mean	SD	
Before	254.1	179.8	283.8	202.2	0.574
During	744.4	518.2	725.4	518.2	0.715
After	401.9	335	423.2	382.6	0.59

*QOL* quality of life, *NRS* numerical rating scale, *HRV* heart rate variability, BF: biofeedback, *J-ZBI* Japanese version of the Zarit Caregiver Burden Interview, *SF-12* 12-Item Short-Form Health Survey, *PCS* physical component summary, *MCS* mental component summary, *RCS* role/social component summary

**Fig. 2:**
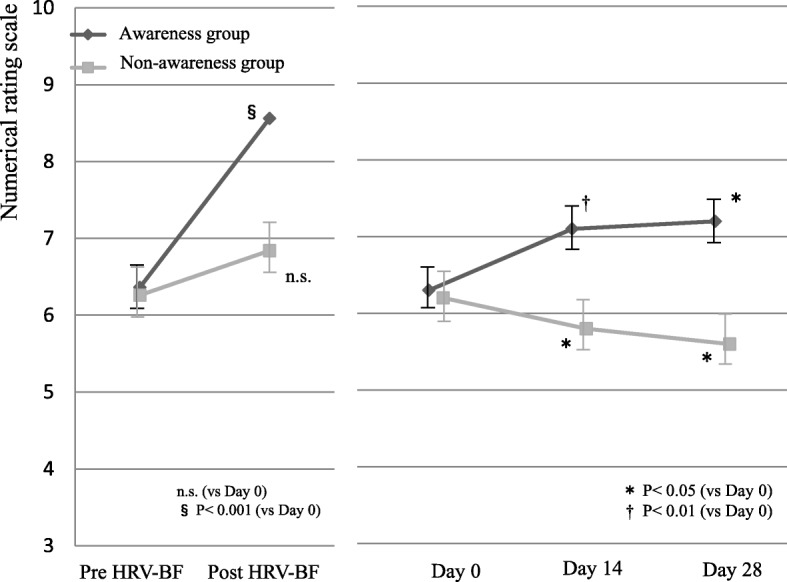
Change in numerical rating scale assessing expectation for treatment from pre and post heart rate variability-biofeedback, and day 0 to day 28. *HRV* heart rate variability, *BF* biofeedback

The therapy expectation levels were assessed using the NRS method. For scores at day 0, there was no significant difference between groups before the therapy session. In contrast, a significant difference was observed between groups after the session (*P* = 0.004) (Table 1). A within-group ANOVA revealed a significant improvement of NRS score in the awareness group after the session at day 0 (*P* < 0.001), but no significant improvement in the non-awareness group (*P* = 0.206). The expectation levels from before the therapy session were significantly increased on days 14 and 28 in the awareness group, but were significantly decreased in the non-awareness group (Fig. 2). No interaction was found between time and group (*P* = 0.129), although a significant main effect of group was identified (*P* = 0.002). A significant interaction between the time and the groups was observed in NRS scores measured before and after the therapy session at day 0 (*P* = 0.004), and a significant intergroup difference was observed regarding the time (*P* = 0.003). Awareness of a feeling of comfort immediately after a therapy session was found to be significantly associated with expectations of the therapy (OR: 12.4; CI, 1.90–81.5) (Table 2).

**Table 2:** Relative factors that influenced the increased expectation for treatment after HRV-BF

Variable	Coefficient β	SE	Wald	*P*-value	OR	95%CI
Age	-0.03	0.04	0.6	0.44	0.97	0.89-1.05
Sex, female	-0.96	0.84	1.3	0.26	0.39	0.74-1.99
Resonant frequency	0.36	0.6	0.35	0.55	1.43	0.44-4.66
Awareness of a feeling of comfort	2.52	0.96	6.9	0.009	12.4	1.90-81.5
J-ZBI	-0.02	0.03	0.39	0.53	0.98	0.92-1.05
LF (day 0, before resonant breathing )	-0.01	0.01	1.07	0.3	0.99	0.98-1.00
LF (day 0, after resonant breathing)	0	0.01	0.07	0.79	1	0.99-1.01

*SE* standard error, *OR* odds ratio, *CI* confidence interval, *J-ZBI* Japanese version of the Zarit Caregiver Burden Interview, *HRV* heart rate variability, *BF* biofeedback

**Fig. 3:**
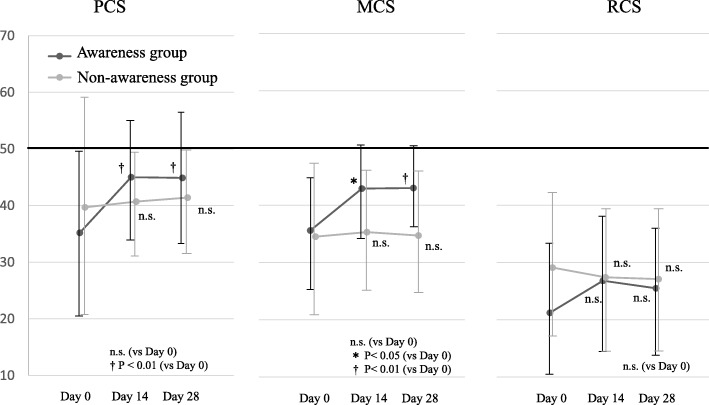
Change in 12-Item Short-Form Health Survey values. *PCS* physical component summary, *MCS* mental component summary, *RCS* role/social component summary, *n.s.* not significant

Scores on MCS and PCS in health-related QOL were significantly increased on days 14 and 28 in the awareness group (Fig. 3). However, we found no significant interaction between the time and the groups in MCS (*P* = 0.220), and no significant main effect of the factor of groups (*P* = 0.560). In addition, the results revealed no interaction between the time and groups in PCS (*P* = 0.183), although the results revealed a significant main effect of groups (*P* = 0.002). No significant change in RCS was observed in either group.

**Fig. 4:**
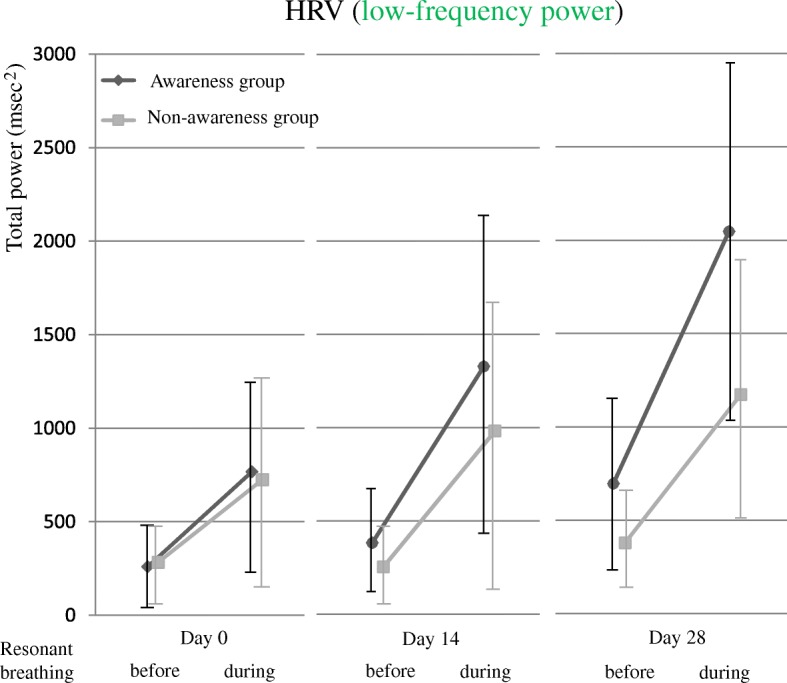
Change in low-frequency values before and during resonant breathing. *HRV* heart rate variability

**Fig. 5:**
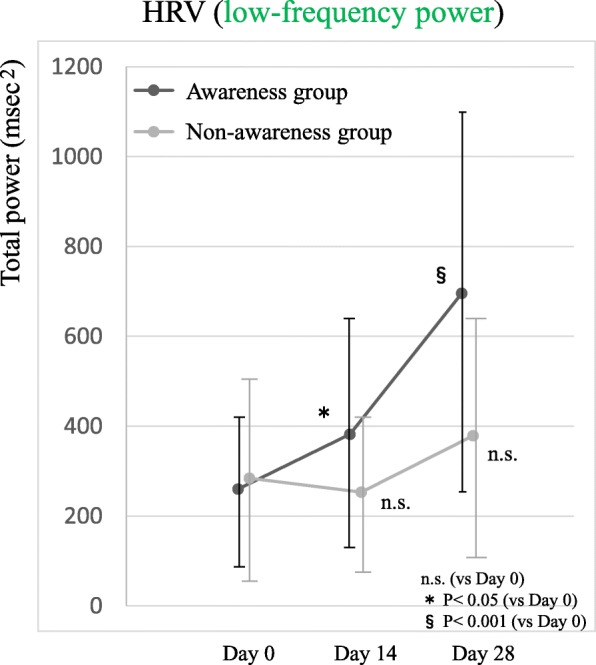
The change in heart rate variability from day 0 to day 28. *HRV* heart rate variability, *n.s.* not significant

Comparison of the low-frequency values before and during resonant breathing revealed no significant difference between groups at day 0 (Table 1), no interaction between the time and the groups (*P* = 0.763), and no main effect of groups (*P* = 0.949) (Fig. 4). At day 28, no interaction was found between the time and the groups (*P* = 0.339), and a main effect of groups was observed in the awareness group (*P* = 0.033). Low-frequency values before resonant breathing increased significantly at days 14 and 28 in the awareness group. Comparison of the low-frequency values between therapy sessions revealed a significant interaction between the time and the groups (*P* = 0.023), and a significant difference between groups regarding the time (day 0: *P* = 0.713, day 14: *P* = 0.190, day 28: *P* = 0.001) (Fig. 5).

## Discussion

The current study had two main findings. First, the feeling of comfort immediately after a relaxation therapy session may have raised participants’ expectations of the therapy. The results indicate that the awareness of a comfortable feeling after a therapy session raised the participants’ expectation levels regarding the therapy and that it was a significant factor associated with the level of expectation. The QOL scores of family caregivers caring for cancer patients are often associated with their self-efficacy [3]. In the current study, initial health-related QOL scores among family caregivers were also decreased, indicating a reduced level of self-efficacy. We propose that, even in this participant population, awareness of feelings of comfort following the relaxation therapy session, which constitutes a coping technique, could lead to increased expectations of the therapy. In terms of other relaxation techniques, a previous single-arm clinical study reported the effectiveness of hypnogenesis for unknown-cause dizziness among advanced cancer patients [26]. In that study, participants were aware of the feeling of psychological change and muscular relaxation, a sign of physiological change in the sleep induction phase. In addition, it was reported that this awareness raised expectations of the therapy and reduced the symptoms of dizziness.

The awareness of the feeling of comfort after a relaxation therapy session could be attributable to an increased awareness of psychological changes in the central nervous system, as well as physiological changes in peripheral tissue with relaxation techniques. Autogenic training is a relaxation technique that is reported to activate cortical function related to emotional experience and interoception [27]. In one model of emotion, the awareness of emotional experience is proposed to activate vagal function, resulting in emotional regulation and stabilization [28]. It has been proposed that a similar process occurs in the awareness of psychological change and peripheral physiological change. In the current study, heart rate variability values increased over time and vagal function was activated in the awareness group.

The second important finding was that low-frequency values before and during resonant breathing at day 0 had no interaction between change process and grouping and no main effect of grouping. This result suggests that the awareness of the feeling of comfort was not associated with the values or changes of HRV during a therapy session and that the improved therapeutic course in the awareness group was not associated with HRV during therapy. In contrast, the awareness of a feeling of comfort immediately after a therapy session raised the level of expectation of the therapy and contributed to a better therapeutic course. These findings indicate that the improved course in the awareness group was associated with the expectation level, which depended on the awareness of a feeling of comfort after a therapy session, but was not associated with HRV during therapy.

It is likely that the participants’ high expectations of the therapy were associated with a placebo effect on the outcome of the therapy. In clinical practice, it has been proposed that placebo effects should be used to a maximum extent to raise patients’ expectations and improve treatment outcomes [29]. In addition, focusing on patients’ individual characteristics, such as therapeutic experience, personality, and genetic factors, has been reported to be useful to maximize therapeutic efficacy [29]. It was found that whether or not patients were aware of a feeling of comfort after a therapy session was dependent on their individual characteristics, and the feeling was considered to play a role in placebo effect, maximizing patients’ expectations of the therapy [29]. Because the main component of the relaxation technique was the patient’s coping ability, the therapy outcomes tended to be influenced by their individual characteristics. One previous study of breast cancer patients reported that the information perceived through their own communication had better effects on future health-related QOL scores than information given by medical professionals [30]. The objective of the therapy in the current study was the acquisition of coping skills. Thus, we hypothesized that expectation levels regarding the therapy would influence the participants’ future behaviors. The current results show the implementation rate of resonant breathing at home to be significantly higher in the awareness group. This high implementation rate could enable the early acquisition of relaxation techniques, which could lead to improvements of QOL and autonomic function.

The current study involved several limitations that should be considered. First, there is no existing data confirming the validity of the Yes/NO answer to the question “Were you aware of a feeling of comfort or discomfort?” to indicate the presence or absence of the awareness of a feeling of comfort or discomfort. This question was intended to evaluate the presence or absence of the awareness of a feeling of comfort, rather than any physiological reaction by a feeling of comfort that the participant was unaware of. NRS assessment of expectation for treatment has often been used in previous studies, but the validity has not been evaluated. Second, no longitudinal assessment was conducted. A previous study of children with anxiety disorder reported that the placebo effect of therapeutic expectation was exerted within 4 weeks after therapy, but was not observed four to 8 weeks after therapy [31]. Third, in the current study, because we conducted a secondary analysis of our previous randomized controlled study, the number of participants performing resonant breathing at home was small and the behavioral effects were not fully evaluated. Fourth, although a number of factors could have affected the expectation levels of the therapy, we were unable to control for confounding factors because of the nature of the prospective observational study design. Finally, because this was a single center study, the generalizability of the study results is limited. Thus, further studies collecting more data are warranted.

## Conclusions

Better outcomes in the awareness group were not associated with HRV during therapy. A feeling of comfort immediately after a therapy session may have positive effects on future QOL and autonomic function by raising the participants’ expectations of the therapy.

## Appendix

### Numerical Rating Scale assessing expectation for treatment

How much do you think your quality of life will improve after this treatment?

Please circle the nearest number.

## Abbreviations

ANOVA: analysis of variance
BF: biofeedback
HRV: heart rate variability
HRV-BF: heart rate variability biofeedback
J-ZBI: Japanese version of the Zarit Caregiver Burden Interview
MCS: mental component summary
NRS: numerical rating scale
PCS: physical component summary
QOL: quality of life
RCS: role/social component summary
SF-12: 12-Item Short-Form Health Survey
